# 2169: Hydrogen bonding and water accessibility changes upon expansion of PolyQ tracts in ataxin-2 and ataxin-3

**DOI:** 10.1017/cts.2017.24

**Published:** 2018-05-10

**Authors:** Jingran Wen, Daniel Scoles, Julio C. Facelli

## Abstract

OBJECTIVES/SPECIFIC AIMS: Polyglutamine (polyQ) neurodegenerative diseases, associated with the unstable expansion of polyQ tracts, are devastating diseases for which no treatments exist. Moreover, most drug discovery attempts have been hindered by the lack of understanding on the relevant pathogenic mechanisms. Here, using previously reported 3D protein predicted structures of ataxin-2 and ataxin-3, we analyze the effect of polyQ enlargement on hydrogen bonding and water accessibility patterns as a possible mechanism for pathogenesis thought enhanced protein aggregation. METHODS/STUDY POPULATION: Using the I-TASSER predicted structures of ataxin-2 and ataxin-3 with different numbers of glutamine repeats representing polyQ lengths characteristic of both normal and pathological tracts (*Journal of Biomolecular Structure and Dynamics*, 2016: 1–16), we identified hydrogen bonds (HBs, UCSF Chimera FindHBond module) and calculated solvent-accessible surface areas (SASA, DSSP program) for the polyQ tracts available in the 3D structures. RESULTS/ANTICIPATED RESULTS: The identified HBs were analyzed as the function of the number of glutamines in the polyQ tracts and characterized as those intra-polyQ and exter-polyQ, respectively. The SASA of the polyQ region was also studied as the function of the polyQ tract length. DISCUSSION/SIGNIFICANCE OF IMPACT: The results obtained here indicate that polyQ regions increasingly prefer self-interactions, which consistently can lead to more compact polyQ structures. The results strongly support the notion that the expansion of the polyQ region can be an intrinsic force leading to self-aggregation of polyglutamine proteins and suggest that the modulation of solvent-polyQ interactions could be a possible therapeutic strategy for polyQ diseases.

